# Anti-Diabetic Effect of Organo-Chalcogen (Sulfur and Selenium) Zinc Complexes with Hydroxy-Pyrone Derivatives on Leptin-Deficient Type 2 Diabetes Model ob/ob Mice

**DOI:** 10.3390/ijms18122647

**Published:** 2017-12-07

**Authors:** Takayuki Nishiguchi, Yutaka Yoshikawa, Hiroyuki Yasui

**Affiliations:** 1 Department of Analytical and Bioinorganic Chemistry, Division of Analytical and Physical Sciences, Kyoto Pharmaceutical University, 5 Nakauchi-cho, Misasagi, Yamashina-ku, Kyoto 607-8414, Japan; kd14006@poppy.kyoto-phu.ac.jp; 2 Department of Health, Sports, and Nutrition, Faculty of Health and Welfare, Kobe Women’s University, 4-7-2 Minatojima-nakamachi, Chuo-ku, Kobe 650-0046, Japan

**Keywords:** diabetes mellitus, organo-chalcogen zinc complexes, ob/ob mice, anti-diabetic effect, inductively coupled plasma mass spectrometry, morphological analysis

## Abstract

Since the discovery of the anti-diabetic effects of zinc (Zn) complex, we synthesized several Zn complexes and evaluated their effects using the KKA^y^ type 2 diabetes mouse model. Recently, we demonstrated that organo-chalcogen (sulfur and selenium) Zn complexes elicit strong anti-diabetic effects. In this study, we treated leptin-deficient ob/ob mice with organo-chalcogen Zn complexes, and evaluated the resulting anti-diabetic effects in a mouse model of diabetes arising from pathogenic mechanisms different from those in KKA^y^ mice. C57BL/6J ob/ob mice orally received either *bis*(3-hydroxy-2-methyl-4(H)-pyran-4-thiono)Zn, [Zn(hmpt)_2_] or *bis*(3-hydroxy-2-methyl-4(H)-pyran-4-seleno)Zn, [Zn(hmps)_2_], daily for 28 days. Both Zn complexes elicited potent blood glucose-lowering effects and improved HbA1c values. Moreover, glucose intolerance improved as evidenced by the oral glucose tolerance test, and fasting plasma insulin levels decreased in both types of Zn complex-treated mice. Zn concentrations in the liver and pancreas of [Zn(hmpt)_2_]-treated mice and in the pancreas of [Zn(hmps)_2_]-treated mice were increased, respectively. The results suggest that the present Zn complexes mainly exerted an anti-diabetic effect in the liver or pancreas. This study is the first to demonstrate that potent Zn complexes elicit anti-diabetic effects in not only KKA^y^ but also ob/ob mice via a normalizing effect on insulin secretion and fasting blood glucose levels.

## 1. Introduction

The number of adults with diabetes mellitus (DM) has been increasing annually and is expected to reach 642 million worldwide by 2040 [1]. DM is generally classified into two main types (1 and 2), with type 2 DM accounting for more than 90–95% of the cases [2]. To treat DM, diet modification, exercise therapy, and administration of various anti-diabetic agents are utilized. However, the agents available for clinical use with DM are associated with problems such as physical and mental discomfort owing to daily insulin injections; furthermore, some of these agents have side effects [3]. Therefore, the development of new types of anti-diabetic agents is essential not only to treat DM but also to improve the quality of life (QOL) in patients with this disorder.

Zinc (Zn) constitutes an essential trace element in humans and plays a pivotal role in health maintenance as a cofactor of several proteins and enzymes [4]. In 1980, Coulston and Dandona reported the first insulin-mimetic activity of Zn ions on rat adipocytes [5]. Subsequently, several research groups have attempted to confirm the insulin-mimetic activity of Zn ion via analysis of the anti-diabetic effects of Zn ions in mouse models of type 1 and type 2 DM [6,7,8].

In our laboratory, we synthesized Zn complexes ([Zn(mal)_2_]) with maltol, which is used as a food additive, and found that the insulin-mimetic activity was higher than that of Zn ion alone [9]. Subsequent to this study, we synthesized and evaluated the anti-diabetic effect of several additional Zn complexes with various ligands, such as picolinic acid, amino acid, and vitamin derivatives [10,11,12]. Overall, we found that Zn complexes with S_2_O_2_ and Se_2_O_2_ coordination modes exhibited the most potent insulin-mimetic activities and anti-diabetic effects [13,14].

In our previous studies, we evaluated the anti-diabetic effect of Zn complexes in vivo using KKA^y^ mice as a model of type 2 DM. KKA^y^ mice are polygenic mice generated by transferring the yellow obese gene (A^y^) into KK mice [15]. KKA^y^ mice spontaneously develop type 2 DM that recapitulates the characteristics of human type 2 DM, such as hyperglycemia, obesity, and insulin resistance. However, a major disadvantage of this model is the lack of an appropriate non-diabetic control. Furthermore, although we have evaluated the anti-diabetic effects of Zn complexes using KKA^y^ mice, few Zn complexes have been evaluated for their anti-diabetic effects using mouse models of type 2 DM arising from different pathogenic mechanisms.

Thus, we consider that it is important to confirm the generality of the anti-diabetic effects of Zn complexes through evaluation using other diabetes mouse models. The ob/ob mouse strain constitutes a genetic leptin-deficient monogenic model of type 2 DM that lacks functional leptin, leading to hyperphagia, obesity, hyperglycemia, hyperinsulinemia, and insulin resistance, thus demonstrating features of human type 2 DM [16]. Therefore, in the present study we evaluated the anti-diabetic effects of Zn complexes, previously confirmed as potent anti-diabetic agents in KKA^y^ mice [17], using the ob/ob type 2 DM mouse model. Our findings indicated that Zn complexes exert potent anti-diabetic effects in ob/ob mice as well as in KKA^y^ mice, and that these likely occur via a normalizing effect on insulin secretion and fasting blood glucose level.

## 2. Results

### 2.1. Syntheses of [Zn(hmpt)_2_] and [Zn(hmps)_2_]

**Figure 1:**
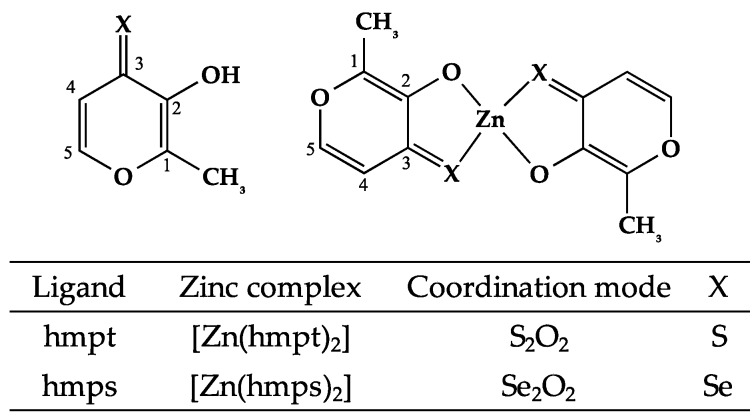
Chemical structures of organo-sulfur (S) and -selenium (Se) zinc (Zn) complexes.

Based on results from elemental analyses and mass spectra of [Zn(hmpt)_2_] and [Zn(hmps)_2_], the Zn complex structure was identified as Zn:ligand at a 1:2 ratio; ^1^H-nucleic magnetic resonance (NMR) spectroscopy yielded hydroxy group (OH) peaks of hmpt and hmps at 7.78 and 7.83 ppm, respectively. However, these peaks were not present in the corresponding Zn complexes, indicating that the hydroxy group was coordinated to the Zn. The ^13^C-NMR data of hmpt and hmps and their corresponding Zn complexes are indicated in Table S1. The peaks for C1, C2 (hydroxy group-linked carbon), and C3 (carbonyl group) were significantly shifted in the complexes compared with those in the corresponding ligands. The downfield shifts in C1 and C2 positions of the Zn complexes were thought to reflect a decrease in electron density in these positions because of a decrease in resonance stabilization by the phenolic hydroxyl group (OH) owing to hydrate group (O^−^) coordination to Zn. In contrast, we considered that the upfield shift of the C3 position was due to an increase in electron density at S and Se atoms by donor-acceptor interaction. In other words, at the polarized bonds generated between Zn of the acceptor atoms and S and Se of the donor atoms, the electron density of the S and Se atoms is increased by a pile-up of electron density at S and Se atoms and spillover of electron density at the Zn atom [18]. These data support a structure wherein these ligands are coordinated to Zn with a hydrate group (C2) and sulfur- and seleno-carbonyl group (C3). From the above results, we confirmed the structures of both [Zn(hmpt)_2_] and [Zn(hmps)_2_] (Figure 1).

### 2.2. In Vitro Insulin-Mimetic Activity Assay

The in vitro insulin-mimetic activity of [Zn(hmpt)_2_] was determined by assessing both free fatty acid (FFA) release-inhibitory and glucose uptake-enhancing effects (Table 1). The activities of both [Zn(hmpt)_2_] and [Zn(hmpt)_2_] were more effective than those of ZnSO_4_ (Zn ion). In addition, the IC_50_ of [Zn(hmpt)_2_] was lower than that of [Zn(hmps)_2_], whereas glucose uptake at 20 μM [Zn(hmps)_2_] was greater than that with [Zn(hmpt)_2_]. Based on the results of in vitro insulin mimetic activities, these complexes were expected to exhibit an anti-diabetic effect in type 2 DM model mice, including in ob/ob mice.

### 2.3. In Vivo Oral Administration of [Zn(hmpt)_2_] and [Zn(hmps)_2_] to ob/ob Mice

**Figure 2:**
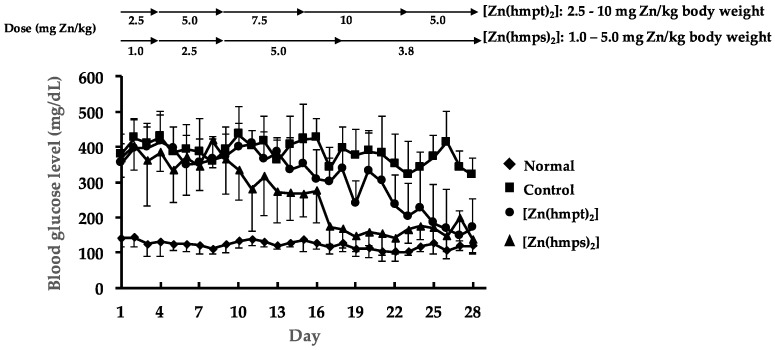
Changes in the daily blood glucose levels over 28 days in normal, control (PEG-400 administered), [Zn(hmpt)_2_]-treated, and [Zn(hmps)_2_]-treated mice; doses of both [Zn(hmpt)_2_] and [Zn(hmps)_2_] are shown in the top part of the figure. Data are expressed as the means ± SDs for 6–8 mice.

We evaluated the anti-diabetic effect of both [Zn(hmpt)_2_] and [Zn(hmps)_2_] in vivo. Both complexes exhibited potent daily blood glucose-lowering effect during oral administration (Figure 2). The blood glucose levels in both [Zn(hmpt)_2_] and [Zn(hmps)_2_]-treated mice were lowered to approximately 200 mg/dL by the end of treatment (Figure 2). Moreover, [Zn(hmps)_2_] exhibited a daily blood glucose-lowering effect at doses lower than those of [Zn(hmpt)_2_].

**Figure 3:**
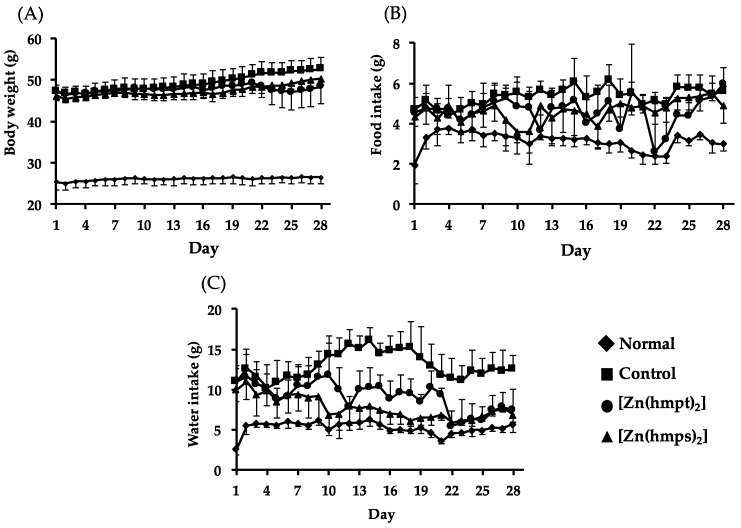
Changes in body weight (**A**); food intake (**B**); and water intake (**C**) over 28 days in normal, control (PEG-400 administered), [Zn(hmpt)_2_]-treated, and [Zn(hmps)_2_]-treated mice; data are expressed as the means ± SDs for 6–8 mice.

Body weight gains in control and [Zn(hmps)_2_]-treated mice were gradually observed. Although body weight loss in [Zn(hmpt)_2_]-treated mice was observed during day 22 to 25 (Figure 3A), the body weight subsequently increased after reduction of the [Zn(hmpt)_2_] dose. It is considered that the body weight loss in [Zn(hmpt)_2_]-treated mice was likely due to decreased food intake caused by gastrointestinal disorder (Figure 3B). In addition, water intake in control mice significantly increased compared with the intake in normal mice, whereas the intake in both [Zn(hmpt)_2_]- and [Zn(hmps)_2_]-treated mice significantly decreased compared with the intake in control mice on the final day (control: 12.6 ± 1.6 g, [Zn(hmpt)_2_]-treated mice: 7.5 ± 2.5 g, and [Zn(hmps)_2_]-treated mice: 6.9 ± 0.5 g. *p* < 0.01 vs. control for each) (Figure 3C). These results indicated that excessive drinking, which is one of the symptoms of DM, improved in the mice treated with either [Zn(hmpt)_2_] or [Zn(hmps)_2_].

**Figure 4:**
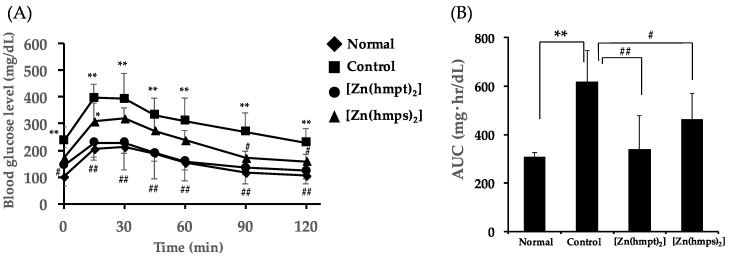
Oral glucose tolerance test (OGTT) after 28 days in normal, control (PEG-400 administered), [Zn(hmpt)_2_]-treated, and [Zn(hmps)_2_]-treated mice. (**A**) Changes in blood glucose levels after gastric gavage of 1 g glucose/kg body weight and (**B**) Area under the blood glucose concentration-time curve (AUC) in OGTT; data are expressed as the means ± SDs for 6–8 mice. Statistical analysis was performed using the Tukey–Kramer test; significance: * *p* < 0.05 and ** *p* < 0.01 vs. normal mice, ^#^ *p* < 0.05 and ^##^ *p* < 0.01 vs. control mice.

We additionally performed oral glucose tolerance tests (OGTT) after oral treatment with Zn complexes for 28 days. Fasting blood glucose levels (the levels in 0 min) in mice treated with either Zn complex decreased, compared with the level in control mice (Control mice: 237 ± 52 mg/dL, [Zn(hmpt)_2_]-treated mice: 143 ± 75 mg/dL (*p* < 0.05 vs. control), and [Zn(hmps)_2_]-treated mice: 172 ± 38 mg/dL, respectively (Figure 4A). We next calculated the area under the blood glucose concentration-time curve (AUC) from the results of changing blood glucose levels after oral administration of 1 g glucose/kg body weight. AUC in both [Zn(hmpt)_2_]-treated and [Zn(hmps)_2_]-treated mice significantly decreased compared with that in control mice (Figure 4B). These results suggested that glucose intolerance improved in the mice treated with either [Zn(hmpt)_2_] or [Zn(hmps)_2_].

Table 2 shows the HbA1c level, which reflects the average blood glucose levels over a long period of time, and plasma parameters after oral treatment with Zn complexes for 28 days. The HbA1c level in control mice significantly increased compared with the level in normal mice, whereas the HbA1c levels in both [Zn(hmpt)_2_] and [Zn(hmps)_2_]-treated mice significantly decreased compared with those in control mice. Plasma insulin levels in control mice were also significantly elevated compared with those in normal mice, whereas both [Zn(hmpt)_2_] and [Zn(hmps)_2_]-treated mice showed reduced levels compared with controls. The results of OGTT, HbA1c, and plasma insulin level assessments suggested that the anti-diabetic effects of the Zn complexes in ob/ob mice were realized owing to improvements in the chronic high glucose state by normalizing insulin secretion. In comparison, plasma adiponectin levels in both [Zn(hmpt)_2_] and [Zn(hmps)_2_]-treated mice did not significantly change compared with the level in control mice.

Both aspartate aminotransferase (AST) and alanine aminotransferase (ALT) levels, which comprise plasma parameters related to hepatic disturbance, were significantly elevated in control compared with normal mice because of fatty liver [19] (Table 2). Both AST and ALT levels in [Zn(hmps)_2_]-treated mice were significantly elevated compared with the levels in control mice, suggesting that hepatic disturbance in [Zn(hmps)_2_]-treated mice may have occurred. Moreover, blood urea nitrogen (BUN) levels in both [Zn(hmpt)_2_] and [Zn(hmps)_2_]-treated mice were significantly elevated compared with control mice, thus suggesting that renal damage in both [Zn(hmpt)_2_] and [Zn(hmps)_2_]-treated mice may have occurred. Triglyceride (TG) and total cholesterol (T-CHO) levels in both [Zn(hmpt)_2_] and [Zn(hmps)_2_]-treated mice were not significantly changed. Alkaline phosphatase (ALP) level in control mice was significantly elevated compared with that in normal mice; however, ALP levels in both [Zn(hmpt)_2_] and [Zn(hmps)_2_]-treated mice significantly decreased compared with the level in control mice.

### 2.4. Determination of Zn and Se Concentrations in the Organs of ob/ob Mice

We investigated the organ distributions of Zn complexes to determine the concentrations of both Zn and Se simultaneously in the organs (plasma, liver, kidney, muscle, pancreas, spleen, and bone) of treated mice using inductively coupled plasma mass spectrometry (ICP-MS). Zn concentrations in the liver significantly increased after treatment with [Zn(hmpt)_2_] (Table 3). In contrast, only Zn concentrations in the plasma significantly increased after oral treatment with [Zn(hmps)_2_].

Table 4 shows the increased concentrations of Δ[Zn] in the organs of [Zn(hmpt)_2_]-treated mice, as compared with control mice, as well as the increased concentrations of Δ[Zn], Δ[Se], and Δ[Zn]/Δ[Se] molar ratios in the organs of [Zn(hmps)_2_]-treated mice. The large increase in Δ[Zn] in the liver and pancreas of [Zn(hmpt)_2_]-treated mice suggested that [Zn(hmpt)_2_] elicits an anti-diabetic effect in these organs (Table 4A). In comparison, because Δ[Zn] only exhibited a large increase in the pancreas of [Zn(hmps)_2_]-treated mice, it was thought that [Zn(hmps)_2_] preferentially induces an anti-diabetic effect in the pancreas (Table 4B).

Prior to oral treatment, the [Zn(hmps)_2_] consisted of Zn and hmps at a molar ratio of 1:2, with a Δ[Zn]/Δ[Se] molar ratio = 0.5. If Zn and the ligand formed [Zn-hmps] in an organ, the complex of Zn and hmps would have a molar ratio of 1:1, with a Δ[Zn]/Δ[Se] molar ratio = 1. We found that the Δ[Zn]/Δ[Se] molar ratios in the plasma and liver were approximately 1.0 and 0.6, respectively. Thus, [Zn(hmps)_2_] in the plasma might exist in [Zn(L)] (L = ligand; hmps) chemical form, whereas in the liver it may exist as a mixture of both [Zn(L)_2_] and [Zn(L)] chemical forms. The ratios in the kidney, bone, and pancreas were 0.25, 6.9, and 27, respectively. These results indicate that dissociated hmps is mainly distributed to the kidney, and that zinc ion (Zn^2+^) dissociated from [Zn(hmps)_2_] is primarily distributed to both the bone and pancreas.

### 2.5. Histopathological Changes in the Pancreas and Liver Following Treatment with Zn Complexes

**Figure 5:**
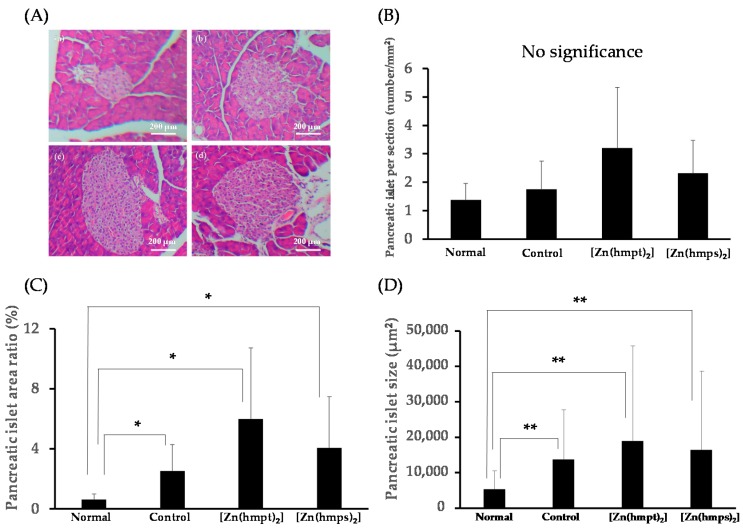
(**A**) Pancreatic morphology in 14-week-old (**a**) normal; (**b**) control (PEG-400 administered); (**c**) [Zn(hmpt)_2_]-treated; and (**d**) [Zn(hmps)_2_]-treated mice. Hematoxylin and eosin staining, ×100 (scale bar = 200 μm); (**B**) Pancreatic islet per section (number/mm^2^) (normal mice: *n* = 57, control mice: *n* = 72, [Zn(hmpt)_2_]-treated mice: *n* = 92, [Zn(hmps)_2_]-treated mice: *n* = 183 pancreatic islets); (**C**) Pancreatic islet area ratio (%); (**D**) Pancreatic islet size (μm^2^). Data are expressed as the means ± SDs for 6–8 mice. Statistical analysis was performed using the Steel-Dwass test; significance: * *p* < 0.05 and ** *p* < 0.01 vs. normal mice.

To evaluate morphological changes in the pancreas and liver, we investigated the effect of both [Zn(hmpt)_2_] and [Zn(hmps)_2_] on pancreatic islet hypertrophy and hepatic glycogen and fat deposition by hematoxylin and eosin (HE) staining, and compared these with the levels in control mice. Figure 5 shows the results of evaluation of pancreatic morphology. Although the pancreatic islet numbers per section among normal, control, and [Zn(hmpt)_2_] and [Zn(hmps)_2_]-treated mice were not significantly changed (Figure 5B), the pancreatic islet area ratio and size in control and [Zn(hmpt)_2_] and [Zn(hmps)_2_]-treated mice significantly increased compared with those of normal mice (Figure 5C,D). These results demonstrate that pancreatic hypertrophy had occurred in control and [Zn(hmpt)_2_] and [Zn(hmps)_2_]-treated mice, indicating that Zn complex treatment did not exert a protective effect on pancreatic islets.

**Figure 6:**
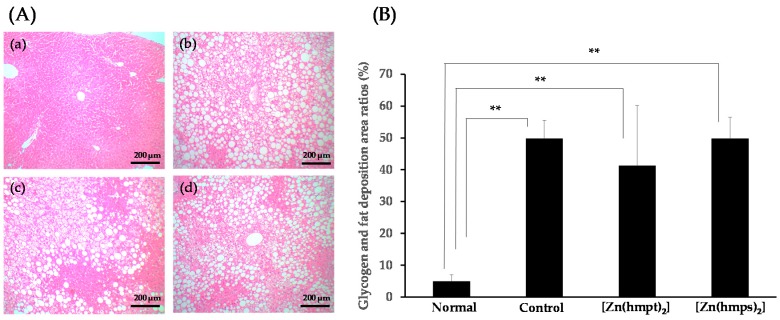
(**A**) Hepatic morphology in 14-week-old normal (**a**); control (PEG-400 administered) (**b**); [Zn(hmpt)_2_]-treated (**c**); and [Zn(hmps)_2_]-treated mice (**d**). Hematoxylin and eosin staining, ×100 (scale bar = 200 μm); (**B**) Glycogen and fat deposition area ratios in the hepatic sectioned tissue of normal, control (PEG-400 administered), [Zn(hmpt)_2_]-treated, and [Zn(hmps)_2_]-treated mice. Data are expressed as the means ± SDs for 6–8 mice. Statistical analysis was performed using the Steel-Dwass test; significance: ** *p* < 0.01 vs. normal mice.

Glycogen and fat deposition area ratios of hepatic sectioned tissue are shown in Figure 6A,B. Excess glycogen and fat deposition in control mice and [Zn(hmpt)_2_] and [Zn(hmps)_2_]-treated mice were observed relative to normal mice. The ratios in control mice, [Zn(hmpt)_2_] and [Zn(hmps)_2_]-treated mice significantly increased compared with those in normal mice (Figure 6B). However, no significant differences in the glycogen and fat deposition area ratio in the liver were observed between control and [Zn(hmpt)_2_]-treated and [Zn(hmps)_2_]-treated mice (Figure 6B).

## 3. Discussion

Several reports investigating the anti-diabetic effect of Zn supplementation in ob/ob mice have been published previously [8,20]. However, to the best of our knowledge, the present study is the first to report the anti-diabetic effect of oral treatment with Zn complexes in ob/ob mice. Our prior studies demonstrated that Zn complexes with hydroxy-pyrone derivatives, including [Zn(hmps)_2_], elicit an anti-diabetic effect in KKA^y^ mice, which constitute a different type of 2 DM model mouse that is accompanied by obesity [17,21]. These anti-diabetic effects of Zn complexes with hydroxy-pyrone derivatives suggest that Zn complexes constitute promising seed compounds for anti-diabetic therapeutics.

Based on the results from in vitro experiments in the present study, marked anti-diabetic effects of both [Zn(hmpt)_2_] and [Zn(hmps)_2_] on ob/ob mice were expected. We tested this hypothesis using the dosages ([Zn(hmpt)_2_]: 2.5–10 mg Zn/kg body weight and [Zn(hmps)_2_]: 1.0–5.0 mg Zn/kg body weight), based on the results of preliminary experiments. [Zn(hmpt)_2_] and [Zn(hmps)_2_] exerted potent daily blood glucose lowering effect during oral treatment (Figure 2). Moreover, [Zn(hmps)_2_] was used at a lower oral dose than the dose (2.0–10 mg Zn/kg body weight) utilized in our previous study with KKA^y^ mice [17]. Our results suggest that Zn complexes with hydroxy-pyrone derivatives may be effective for type 2 DM caused by insulin resistance based on obesity. In contrast, two adverse effects of treatment with Zn complexes occurred in the present study. The first was body weight loss in [Zn(hmpt)_2_]-treated mice. Zn is a relative safe trace element; however, the tendency of excess Zn intake to induce gastrointestinal disorder has been well documented [22]. It was therefore considered that the body weight loss in [Zn(hmpt)_2_]-treated mice might be caused by gastrointestinal disorder based on excess Zn intake.

The second adverse effect comprised liver disturbance in [Zn(hmps)_2_]-treated mice, as suggested by the observed increase of AST and ALT levels in these animals (Table 2). Conversely, glycogen and lipid deposition area ratios in the liver following [Zn(hmps)_2_] treatment were not significantly altered compared with those of control mice (Figure 6A,B). Further, as shown in Table S2, the deposition area may consist of TG. These results suggested that the liver disturbance was not an indirect result of the promotion of glycogen and fat accumulation but rather a direct disturbance by [Zn(hmps)_2_] or hmps. Moreover, it has been reported that necrotic areas in hepatic sections were observed in male Wistar rats fed food containing sodium selenite, which is an inorganic selenium compound (10 µg sodium selenite/kg/day, 4.6 µg Se/kg/day), for 3 months [23]. Furthermore, it was also reported that 100% lethality and vacuolation of centrilobular and peripheral hepatocytes were observed in male ICR mice orally administered d,l-selenocysteine, an organic selenium compound, once daily (30 mg d,l-selenocysteine/kg/day, 13.9 mg Se/kg/day), six days per week, for 30 days [24]. Taking these results into consideration, we speculate that the liver disturbance was caused by hmps.

From the results of Δ[Zn] in both [Zn(hmpt)_2_] and [Zn(hmps)_2_]-treated mice, it was thought that [Zn(hmpt)_2_] or [Zn(hmps)_2_] might exhibit an anti-diabetic effect in both the liver and the pancreas or the pancreas, respectively. In the liver of ob/ob mice, it has been reported that both a decrease in glycogen synthesis and an increase in gluconeogenesis caused by insulin-resistance occurred [25,26]. Moreover, Zn ion has been shown to exhibit an anti-diabetic effect by inhibiting phosphatases, which negatively regulate insulin signaling cascades, such as protein tyrosine phosphatase 1B (PTP-1B) and phosphatase and tensin homolog deleted from chromosome 10 (PTEN) [27]. In addition, we reported that [Zn(hkt)_2_] (hkt; hinokitiol) might exert an anti-diabetic effect in KKA^y^ mice to improve insulin sensitivity by inhibiting both PTP-1B and PTEN [28,29]. In comparison, another study found that in HepG2 cells, Zn ion enhanced the phosphorylation of FoxO1a, which is related to gluconeogenesis, by activation of the PI3-Akt pathway in the insulin signaling cascade, during which FoxO1a is transferred from the nucleus to the cytoplasm [30]. Thus, [Zn(hmpt)_2_] might exhibit an anti-diabetic effect by the same mechanism in the liver of ob/ob mice. Therefore, we considered that investigating the influence of Zn complexes on gluconeogenesis-related-genes and the corresponding enzyme activities, such as phosphoenolpyruvate carboxykinase (PEPCK) or glucose-6-phosphatase (G6Pase), is required in future studies.

In addition, it has recently been reported that Zn, which is expelled together with insulin from the pancreas, protects insulin from degradation owing to its uptake into hepatic cells [31]. Thus, it is thought that as both Zn complexes showed an anti-diabetic effect in the pancreas, [Zn(hmpt)_2_] and [Zn(hmps)_2_] may each have supplied Zn ions to the pancreas and might exhibit an anti-diabetic effect owing to enhancement of peripheral insulin utilization through inhibition of the insulin degradation in the liver. This supports the potential utility of these compounds as the basis for developing effective therapeutics for type 2 DM targeting the pancreas.

In our previous study, preservation effects on the pancreas using Zn complexes, including [Zn(hmps)_2_], were observed in KKA^y^ mice [17]. In ob/ob mice, because insulin section is increased to compensate for the peripheral insulin insufficiency caused by insulin resistance, these animals develop hyperinsulinemia compared with normal +/+ mice. The mechanism of increased insulin secretion has been shown to be caused by increases of β cells resulting from hyperplasia of pancreatic islet [32]. Thus, in the present study, we expected that the islet area in both [Zn(hmpt)_2_] and [Zn(hmps)_2_]-treated mice would be smaller than that in control mice. However, although significant differences of the islet area among control, [Zn(hmpt)_2_], and [Zn(hmps)_2_]-treated mice were not observed, the areas in both [Zn(hmpt)_2_] and [Zn(hmps)_2_]-treated mice tended to be larger than that in control mice (Figure 5). Insulin signaling in pancreatic β cells is known to play an important role in the enlargement of islets [33,34]. Alternatively, Zn ion and Zn complexes improve DM to enhance insulin signaling [28,29]. Moreover, the results of ICP-MS indicated that although significant differences were not observed, pancreatic Zn concentrations in [Zn(hmpt)_2_] and [Zn(hmps)_2_]-treated mice tended to increase compared to those in control mice (Table 3). Thus, an enlargement of the islet area may have occurred owing to an increase in β cells mediated by enhancement of insulin signaling by Zn ions in the pancreas. Furthermore, we reported that [Zn(hkt)_2_] increased pancreatic duodenal homeobox-1 (PDX-1), which is a transcription factor that translocates into the nucleus and induces the transcription of various gene including insulin, glucokinase (GK) and glucose transporter type 2 (GLUT 2) [35], as well as mRNA expression in RIN-5F cells, which are insulinoma β cells derived from rat [29]. It has also been reported that insulin productive cells (i.e., β cells) are newly produced by overexpression of PDX-1 in the pancreas via adenovirus vector [36]. Together, these observations suggest that the islet hypertrophy in both [Zn(hmpt)_2_] and [Zn(hmps)_2_]-treated mice may also have occurred through enhancing the increase in β cells owing to increased PDX-1 expression in the pancreas.

In contrast, the fasting plasma insulin levels in both [Zn(hmpt)_2_] and [Zn(hmps)_2_]-treated mice decreased compared to the levels in control mice (Table 2). Considering the results regarding the characteristics of the anti-diabetic effect including lowered daily blood glucose and improvements of HbA1c and glucose intolerance in OGTT, although islet hypertrophy may have occurred by treatment with both [Zn(hmpt)_2_] and [Zn(hmps)_2_], it is likely that hyperinsulinemia did not occur in neither [Zn(hmpt)_2_] nor [Zn(hmps)_2_]-treated mice owing to the normalization of insulin secretion.

## 4. Materials and Methods

### 4.1. Chemicals

Zinc sulfate heptahydrate (ZnSO_4_·7H_2_O), lithium hydroxide monohydride (LiOH·H_2_O), and polyethylene glycol 400 (PEG-400) were obtained from Wako Pure Chemicals Co. (Osaka, Japan); 3-Hydroxy-2-methyl-4(H)-pyran-4-one (maltol) was from Tokyo Chemical Industry Co. (Tokyo, Japan). Lawesson’s reagent, (±)-adrenaline hydrochloride, and bovine serum albumin (BSA: fraction V) were obtained from Sigma Aldrich Inc. (St. Louis, MO, USA). Dry toluene and dichloromethane were obtained from Nacalai Tesque Inc. (Kyoto, Japan). Acetone was obtained from JUNSEI Chemical Co., Ltd. (Kyoto, Japan). Dimethyl sulfoxide was obtained from Kishida Chemical Co. (Osaka, Japan).

### 4.2. Analytical Instrumentation

Elemental analyses were performed using a Perkin-Elmer 240CHN elemental analyzer (PerkinElmer Japan Co., Ltd., Tokyo, Japan). Mass spectra were recorded by a JEOL JMS-SX 102 AQQ mass spectrometer (JEOL Ltd., Tokyo, Japan). ^1^H- and ^13^C-NMR were measured using a Varian Unity Inova spectrometer (Varian, Inc., Palo Alto, CA, USA), with tetramethylsilane as an internal standard.

### 4.3. Experimental Animals

Male Wistar rats (8 weeks old), type 2 diabetic ob/ob mice (8 weeks old, *n* = 30), and non-diabetic +/+ mice (8 weeks old, *n* = 6) were purchased from Shimizu Experimental Laboratory Inc. (Kyoto, Japan). The ob/ob and +/+ mice were used for in vivo studies when they were 10 weeks old. They were housed in an air-conditioned room at a temperature of 23 ± 1 °C and a humidity of 60 ± 10%, with lights on from 8:00 to 20:00. The animal studies were approved by the Bioscience Research Center at the Kyoto Pharmaceutical University (KPU) and performed according to the Guidelines for Animal Experimentation at the KPU. The number and detailed date of this approval for animal studies were 15-13-013 and 31 March 2015, respectively.

### 4.4. Syntheses of [Zn(hmpt)_2_] and [Zn(hmps)_2_]

#### 4.4.1. HMPT: 3-Hydroxy-2-Methyl-4(H)-Pyran-4-Thione

Lawesson’s reagent was added to a hot solution of maltol in dry toluene at 80 °C under nitrogen gas. The resulting solution was heated with stirring for 1.5 h, cooled to room temperature (22–25 °C), and clarified by filtration. The filtrate was evaporated to yield a dark orange oil that was purified using a silica gel column (4.0–6.3 μm mesh, dichloromethane solvent). After evaporation of the solvent, the product was obtained as a yellow solid substance (yield: 53%). Anal. found (Calcd.) for C_6_H_6_O_2_S: C, 50.70 (50.68); H, 4.02 (4.25). EI(+)-MS: *m*/*z* 142 [M+]. ^1^H-NMR (400 MHz, CDCl_3_): δ_H_ 7.78 (s, 1H), 7.58 (d, *J* = 5.04 Hz, 1H), 7.32 (d, *J* = 4.99 Hz, 1H), 2.45 (s, 3H). ^13^C-NMR (100 MHz, CDCl_3_): δ_C_ 185.88, 150.61, 146.97, 145.27, 124.20, 15.07.

#### 4.4.2. HMPS: 3-Hydroxy-2-Methyl-4(H)-Pyran-4-Selenone [17]

Woollins’ reagent was added to a hot solution of maltol in dry toluene at 80 °C under nitrogen gas. The resulting solution was heated with stirring for 12 h, cooled to room temperature, and clarified by filtration. The filtrate was evaporated to yield a dark red oil that was purified by silica gel column (4.0–6.3 μm mesh, dichloromethane solvent). The oil cooled in freezer was converted into a dark red solid substance (yield: 57%). Anal. found (Calcd.) for C_6_H_6_O_2_Se: C, 38.11 (38.11); H, 2.98 (3.20). EI(+)-MS: *m*/*z* 190 [M+]. ^1^H-NMR (400 MHz, CDCl_3_): δ_H_ 7.83 (s, 1H), 7.68 (d, *J* = 4.90 Hz, 1H), 7.56 (d, *J* = 4.90 Hz, 1H), 2.32 (s, 3H). ^13^C-NMR (100 MHz, CDCl_3_): δ_C_ 185.83, 154.21, 145.49, 145.03, 129.55, 15.63.

#### 4.4.3. [Zn(hmpt)_2_]: *bis*(3-Hydroxy-2-Methyl-4(H)-Pyran-4-Thiono)Zn

A methanol solution of LiOH·H_2_O was added to a solution of hmpt, followed by stirring for 30 min at room temperature under nitrogen gas. After 30 min, a methanol solution of ZnSO_4_·7H_2_O was added to the mixture, followed by stirring for 2.5 h under nitrogen gas. After removing the solvent, the obtained yellow precipitate was recrystallized with acetone (yield: 32%). Anal. found (Calcd.) for C_12_H_10_O_4_S_2_Zn: C, 41.41 (41.44); H, 2.62 (2.90). EI(+)-MS: *m*/*z* 346 [M+]. ^1^H-NMR (400 MHz, CDCl_3_): δ_H_ 7.72 (d, *J* = 4.58 Hz, 1H), 7.59 (d, *J* = 4.58 Hz, 1H), 2.62 (s, 3H). ^13^C-NMR (100 MHz, CDCl_3_): δ_C_ 176.83, 159.99, 154.23, 144.60, 122.54, 16.64.

#### 4.4.4. [Zn(hmps)_2_]: *bis*(3-Hydroxy-2-Methyl-4(H)-Pyran-4-Seleno)Zn [17]

A methanol solution of ZnSO_4_·7H_2_O was added to a solution of hmps, followed by stirring for 5 min at room temperature under nitrogen gas. After 5 min, a methanol solution of LiOH·H_2_O was added to the mixture, followed by stirring for 1 h under nitrogen gas. After removing the solvent, the obtained orange precipitate was washed with cold methanol (yield: 89%). Anal. found (Calcd.) for C_12_H_10_O_4_Se_2_Zn: C, 32.72 (32.64); H, 2.15 (2.28). EI(+)-MS: *m*/*z* 442 [M+]. ^1^H-NMR (400 MHz, CDCl_3_): 7.88 (d, *J* = 4.53 Hz, 1H), 7.63 (d, *J* = 4.49 Hz, 1H), 2.57 (s, 3H). ^13^C-NMR (100 MHz, CDCl_3_): δ_C_ 175.17, 162.38, 154.42, 143.27, 126.19, 17.18.

### 4.5. In Vitro Insulin Mimetic Activity Assay

The in vitro insulin-mimetic activities of Zn complexes were determined by the inhibition of FFA release from adipocytes and increase of glucose uptake into adipocytes [37]. Isolated male Wistar rat epididymal adipocytes (1.0 × 10^6^ cells/mL) were prepared and pre-incubated separately with various concentrations of the different Zn complexes in 5 mM glucose in Krebs-Ringer bicarbonate buffer (120 mM NaCl, 1.3 mM CaCl_2_, 1.2 mM MgSO_4_, 4.8 mM KCl, 1.2 mM KH_2_PO_4_, 24 mM NaHCO_3_, pH 7.4) containing 2% BSA, with shaking at 100 cycles/min for 30 min at 37 °C. Adrenaline (15 μL, 0.2 mM) was then added to the mixtures, and the resultant solutions were then incubated for 3 h at 37 °C. The reaction mixture was subsequently centrifuged at 650× *g* for 10 min at 4 °C. FFA levels in the extracellular solutions were determined using an FFA measuring kit (NEFA C-test Wako) and the IC_50_ value, which is the 50% inhibitory concentration of FFA-release in each complex, was calculated. Glucose concentrations in the solutions with 20 μM Zn complexes were determined using a Fuji Dry Chem system (Fuji Medical Co., Tokyo, Japan) and then the glucose uptake quantities/(1.0 × 10^6^ cells) in the 20 μM Zn complexes were calculated.

### 4.6. In Vivo Oral Treatment with both [Zn(hmpt)_2_] and [Zn(hmps)_2_] of ob/ob Mice

The anti-diabetic effects of both [Zn(hmpt)_2_] and [Zn(hmps)_2_] were examined in ob/ob mice. The ob/ob and +/+ mice were used as type 2 diabetic and normal (non-diabetic) mice, respectively. The ob/ob (10-week-old) and +/+ (10-week-old) mice received daily oral treatment of either Zn complexes dissolved in PEG-400 or PEG-400 only, respectively. The mice received oral treatments of [Zn(hmpt)_2_] or [Zn(hmps)_2_] at doses of 2.5–10 mg Zn/kg or 1.0–5.0 mg Zn/kg for 28 days, respectively. Blood glucose levels (BGL) were measured using a Glucocard (Arkray, Kyoto, Japan) just before treatment with the Zn complexes, and the doses were regulated based on the BGL. Over 28 days, BGL, body weight, food intake, and water intake were measured every day. The number of normal mice (+/+ mice), control mice (ob/ob mice), [Zn(hmpt)_2_]-treated, and [Zn(hmps)_2_]-treated mice was 6, 8, 8, and 8 in the initial animal experiments, respectively. This was modified to 6, 6, 7, and 8 after the end of experiments as two control mice died naturally by the end of experiments, and one [Zn(hmpt)_2_]-treated mouse died from an accident during the oral glucose tolerance test.

### 4.7. OGTT to ob/ob Mice after Treatment with Zn Complexes

After treatment with [Zn(hmpt)_2_] or [Zn(hmps)_2_] for 28 days, OGTTs were performed. The ob/ob mice and +/+ mice were fasted for 12-h and glucose (1 g/kg body weight) was given orally. Blood samples were obtained from the tail vein at 0, 15, 30, 45, 60, 90, and 120 min after glucose administration. Blood glucose concentrations were measured using a Glucocard.

### 4.8. Determination of Blood Parameters and Organ Distributions of Zn and Se in ob/ob Mice after Treatment with Zn Complexes

After OGTT, HbA1c levels were measured in blood obtained from the mouse tail vein using an immunoassay with a DCA 2000 analyzer (Bayer-Sankyo Co., Ltd., Tokyo, Japan). The mice were subjected to a 12-h fast and blood samples were collected from the orbital vein or postcaval vein under isoflurane anesthesia using heparinized tools. Collected samples were then centrifuged at 650× *g* for 10 min at 4 °C, and the resultant plasma samples were used to analyze various biochemical parameters. The levels of AST, ALT, BUN, TG, T-CHO, and ALP in plasma samples were determined using a Fuji Dry Chem system. The plasma levels of insulin and adiponectin were determined using an ultra-sensitive Mouse Insulin ELISA Kit (Morinaga Institute of Biological Science, Inc., Kanagawa, Japan) and an adiponectin immunoassay kit (R&D Systems Inc., Minneapolis, MN, USA), respectively. After collecting the blood samples, mice were subjected to dissection under isoflurane anesthesia, and the resultant tissue samples were washed in saline. Each of the samples was dehydrated for a week in desiccators, weighed, and then heated in 5% HNO_3_ (5 mL) for 5 h at 110 °C in a closed system. The resultant solution was centrifuged at 1250× *g* for 15 min, and the supernatant was diluted using 5% HNO_3_. Both Zn and Se concentrations in organs were determined with an ICP-MS Agilent 7700x/Mass Hunter instrument (Agilent Technologies Inc., Santa Clara, CA, USA). The Zn and Se concentrations in each sample were calculated using linear regression of the standard curves prepared from standard solutions of the respective trace elements.

### 4.9. Histopathology and Morphometric Analysis

Excised pancreas and liver from 14-week-old mice were immediately fixed in 10% buffered formalin for a week, sectioned at a thickness of 3 μm, and stained with HE for histopathological examination. HE-stained tissue sections were scanned using a Moticam T2 (Shimadzu Co., Kyoto, Japan) to prepare digital images. Both pancreatic islet area and glycogen and fat deposition areas were calculated with the dedicated image processing software WinROOF (Mitani Corp., Tokyo, Japan).

### 4.10. Detection of Glycogen and TG in the Liver

Lipids in the liver were extracted according to modified Folch method [38]. TG in the extracted lipids was detected using the triglyceride E test wako (Wako Pure Chemicals Co., Osaka, Japan). Glycogen in the liver was extracted with a strong base (30% KOH aq). Then, ethanol precipitation methods (using 95% ethanol and saturated Na_2_SO_4_), hydrolysis, and neutralization (with 2.4 M HCl aq and 0.8 M NaOH aq) of the glycogen extracts were performed, resulting in degradation to glucose solution. To detect the glucose levels in the solution using the glucose CII test wako (Wako Pure Chemicals Co., Osaka, Japan), glycogen levels in the liver were estimated. Because the liver sample of normal mice was used up during TG detection and ICP-MS analysis, glycogen content in the liver could not be detected.

### 4.11. Statistical Analysis

Data are expressed as the means ± SDs. Significant differences among groups were analyzed by one-way analysis of variance and Tukey–Kramer, or Steel-Dwass multiple comparison tests. Differences were considered significant at *p* < 0.05 or *p* < 0.01.

## 5. Conclusions

In this study, it was found that Zn complexes with hydroxy-pyrone derivatives elicited an anti-diabetic effect in leptin-deficient ob/ob mice, as previously shown for KKA^y^ mice, via a normalizing effect on insulin secretion and fasting blood glucose level. Moreover, the Zn and Se concentrations in each organ, as determined by ICP-MS, indicated that [Zn(hmpt)_2_] induces anti-diabetic effects in the liver and pancreas, whereas [Zn(hmps)_2_] may exert its effects in the pancreas. Although further research is needed, the findings regarding the Zn complexes used in this study support these as suitable seed compounds for anti-diabetic therapeutics.

## Figures and Tables

**Table 1:** IC_50_ values of free fatty acid release and glucose uptake using 20 μM Zn complexes.

Zn Complex	IC_50_ (μM)	Glucose Uptake (µg/10^6^ Cells)
[Zn(hmpt)_2_]	2 ± 1	79 ± 26
[Zn(hmps)_2_] [17]	8 ± 1	138 ± 6
ZnSO_4_ [17]	585 ± 305	none

Data are expressed as the means ± SDs for three runs.

**Table 2:** HbA1c levels and plasma parameters in normal, control (PEG-400 administered), [Zn(hmpt)_2_]-treated and [Zn(hmps)_2_]-treated mice.

	Normal	Control	[Zn(hmpt)_2_]	[Zn(hmps)_2_]
HbA1c (%)	4.1 ± 0.3	7.9 ± 0.8 **	6.5 ± 0.7 **^,##^	6.7 ± 0.4 **^,##^
Insulin (ng/mL)	0.3 ± 0.0	2.2 ± 1.0 **	0.9 ± 0.3 ^##^	1.4 ± 0.6 **
Adiponectin (µg/mL)	12.8 ± 2.7	8.2 ± 1.1 **	9.4 ± 0.8 **	9.0 ± 0.8 **
AST (U/L)	34 ± 6	93 ± 19 *	103 ± 25 **	200 ± 55 **^,##,††^
ALT (U/L)	13 ± 2	164 ± 64 *	118 ± 68	355 ± 138 **^,##,††^
BUN (mg/dL)	16 ± 1	17 ± 4	23 ± 2 **^,##^	24 ± 3 **^,##^
TG (mg/dL)	30 ± 3	83 ± 16 **	76 ± 25 **	87 ± 11 **
T-CHO (mg/dL)	76 ± 12	195 ± 16 **	189 ± 42 **	186 ± 16 **
ALP (U/L)	257 ± 28	723 ± 126 **	360 ± 88 ^##^	550 ± 89 **^,##,††^

Data are expressed as the means ± SDs for 6–8 mice; statistical analysis was performed using a Tukey-Kramer test; significance: * *p* < 0.05 and ** *p* < 0.01 vs. normal mice, ^##^ *p* < 0.01 vs. control mice, ^††^ *p* < 0.01 vs. [Zn(hmpt)_2_]-treated mice. AST, aspartate aminotransferase; ALT, alanine aminotransferase; BUN, blood urea nitrogen; TG, triglyceride; T-CHO, total cholesterol; ALP, alkaline phosphatase.

**Table 3:** Zn and Se concentrations in dry tissues (µg/g) and plasma (µg/mL) in normal, control (PEG-400 administered), [Zn(hmpt)_2_]-treated, and [Zn(hmps)_2_]-treated mice.

Organ	Zn				Se			
	Normal	Control	[Zn(hmpt)_2_]	[Zn(hmps)_2_]	Normal	Control	[Zn(hmpt)_2_]	[Zn(hmps)_2_]
Plasma	1.8 ± 0.2	2.1 ± 0.1	2.3 ± 0.2 **	2.5 ± 0.2 **^,##^	0.4 ± 0.1	0.6 ± 0.1 *	0.5 ± 0.1	1.1 ± 0.2 **^,##,††^
Liver	99 ± 10	44 ± 5 **	74 ± 30 ^#^	46 ± 6 ^##^^,†^	5.4 ± 0.6	2.1 ± 0.3 **	2.4 ± 0.7 **	5.1 ± 0.6 ^##,††^
Kidney	77 ± 6	67 ± 3 **	73 ± 4	68 ± 4 **	6.6 ± 0.2	6.4 ± 0.2	5.8 ± 0.5	12.7 ± 2.0 **^,##,††^
Muscle	44 ± 6	47 ±10	45 ± 8	41 ± 2	1.2 ± 0.4	1.3 ± 0.4	1.2 ± 0.2	2.5 ± 0.4 **^,#,††^
Pancreas	121 ± 14	133 ± 19	153 ± 18	158 ± 28 *	1.7 ± 0.5	2.3 ± 0.6	1.6 ± 0.9	3.4 ± 0.8 **^,##,††^
Spleen	87 ± 9	100 ± 17	95 ± 11	95 ± 12	2.4 ± 0.9	2.7 ± 0.8	2.2 ± 0.3	9.1 ± 2.4 **^,##,††^
Bone	177 ± 8	154 ± 7 **	188 ± 8 ^##^	162 ± 10 *^,^^††^	0.6 ± 0.3	0.5 ± 0.2	0.5 ± 0.2	1.9 ± 0.6

Data are expressed as the means ± SDs for 4–8 mice; statistical analysis was performed using the Tukey-Kramer test; significance: * *p* < 0.05 and ** *p* < 0.01 vs. normal mice, ^#^ *p* < 0.05 and ^##^ *p* < 0.01 vs. control mice, ^†^ *p* < 0.05 and ^††^ *p* < 0.01 vs. [Zn(hmpt)_2_]-treated mice.

**Table 4:** Increased concentrations of Zn, Δ[Zn] (nmol/g), in organs of [Zn(hmpt)_2_]-treated mice compared with control mice (**A**), and increased concentrations of Zn and Se, Δ[Zn] (nmol/g) and Δ[Se] (nmol/g), and Δ[Zn]/Δ[Se] molar ratios, in organs of [Zn(hmps)_2_]-treated mice compared with control mice (**B**).

**(A)**					
	**Plasma**	**Liver**	**Kidney**	**Bone**	**Pancreas**
Δ[Zn] (nmol/g)	3	453	86	517	302
**(B)**					
	**Plasma**	**Liver**	**Kidney**	**Bone**	**Pancreas**
Δ[Zn] (nmol/g)	6	23	20	124	375
Δ[Se] (nmol/g)	6	38	81	18	14
Δ[Zn]/Δ[Se]	1.0	0.61	0.25	6.9	27

[Zn(L)]: Δ[Zn]/Δ[Se] = 1.0, [Zn(L)_2_]: Δ[Zn]/Δ[Se] = 0.5.

## Supplementary Materials

Supplementary materials can be found at www.mdpi.com/1422-0067/18/12/2647/s1.

## Abbreviations

ALP	alkaline phosphatase
AST	aspartate aminotransferase
ALT	alanine aminotransferase
BUN	blood urea nitrogen
G6Pase	glucose-6-phosphatase
GK	glucokinase
GLUT2	glucose transporter type 2
HE	hematoxylin and eosin
hmps	3-hydroxy-2-methyl-4(H)-pyran-4-selenone
hmpt	3-hydroxy-2-methyl-4(H)-pyran-4-thione
ICP-MS	inductively coupled plasma mass spectrometry
OGTT	oral glucose tolerance test
PDX-1	pancreatic duodenal homeobox-1
PEPCK	phosphoenolpyruvate carboxykinase
PTEN	phosphatase and tensin homolog deleted from chromosome 10
PTP-1B	protein tyrosine phosphatase 1b
T-CHO	total cholesterol
TG	triglyceride
[Zn(hmps)_2_]	*bis*(3-hydroxy-2-methyl-4(H)-pyran-4-seleno)Zn
[Zn(hmpt)_2_]	*bis*(3-hydroxy-2-methyl-4(H)-pyran-4-thiono)Zn
