# Supplementary material for: Anti-Diabetic Effect of Organo-Chalcogen (Sulfur and Selenium) Zinc Complexes with Hydroxy-Pyrone Derivatives on Leptin-Deficient Type 2 Diabetes Model ob/ob Mice
Source: Int J Mol Sci. 2017 Dec 7;18(12):2647. doi: 10.3390/ijms18122647 (PMC5751249; doi:10.3390/ijms18122647)
Supplement: Supplementary file 1 [file ijms-18-02647-s001.pdf]

Table S1. ^{13}C-NMR chemical shift (Δδ) data for organo–sulfur (S) and selenium (Se) ligands and the corresponding Zn complexes in $CDCl_3$.

Compound	Chemical shifts (ppm)				
	C1	C2	C3	C4	C5
hmpt	145.27	150.61	185.88	124.20	146.97
[Zn(hmpt)$_2$]	154.23	159.99	176.83	122.54	144.60
Δδ	+8.96	+9.38	-9.05	-1.66	-2.37
hmps [17]	145.03	154.21	185.83	129.55	145.49
[Zn(hmps)$_2$] [17]	154.40	162.38	175.17	126.19	143.27
Δδ [17]	+9.39	+8.17	-10.66	-3.36	-2.22

Table S2. Triglyceride and glycogen contents in the liver of ob/ob mice treated with Zn complexes.

	Normal	Control	[Zn(hmpt)$_2$]	[Zn(hmps)$_2$]
TG (mg/g liver)	16 ± 11	437 ± 139	309 ± 134	443 ± 42
Glycogen (mg/g liver)	-	0.08 ± 0.04	0.03 ± 0.01	0.04 ± 0.01

Data are expressed as mean ± SDs for 6–8 mice. The detailed methods for measuring both TG and glycogen contents in the liver are described in Materials and Methods section (4.10).
